# Scale-Invariant Neuronal Avalanche Dynamics and the Cut-Off in Size Distributions

**DOI:** 10.1371/journal.pone.0099761

**Published:** 2014-06-13

**Authors:** Shan Yu, Andreas Klaus, Hongdian Yang, Dietmar Plenz

**Affiliations:** 1 Section of Critical Brain Dynamics, National Institute of Mental Health, Bethesda, Maryland, United States of America; 2 Nobel Institute for Neurophysiology, Department of Neuroscience, Karolinska Institute, Stockholm, Sweden; National Scientific and Technical Research Council (CONICET), Argentina

## Abstract

Identification of cortical dynamics strongly benefits from the simultaneous recording of as many neurons as possible. Yet current technologies provide only incomplete access to the mammalian cortex from which adequate conclusions about dynamics need to be derived. Here, we identify constraints introduced by sub-sampling with a limited number of electrodes, i.e. spatial ‘windowing’, for well-characterized critical dynamics―neuronal avalanches. The local field potential (LFP) was recorded from premotor and prefrontal cortices in two awake macaque monkeys during rest using chronically implanted 96-microelectrode arrays. Negative deflections in the LFP (nLFP) were identified on the full as well as compact sub-regions of the array quantified by the number of electrodes *N* (10–95), i.e., the window size. Spatiotemporal nLFP clusters organized as neuronal avalanches, i.e., the probability in cluster size, *p*(*s*), invariably followed a power law with exponent −1.5 up to *N*, beyond which *p*(*s*) declined more steeply producing a ‘cut-off’ that varied with *N* and the LFP filter parameters. Clusters of size *s*≤*N* consisted mainly of nLFPs from unique, non-repeated cortical sites, emerged from local propagation between nearby sites, and carried spatial information about cluster organization. In contrast, clusters of size *s*>*N* were dominated by repeated site activations and carried little spatial information, reflecting greatly distorted sampling conditions. Our findings were confirmed in a neuron-electrode network model. Thus, avalanche analysis needs to be constrained to the size of the observation window to reveal the underlying scale-invariant organization produced by locally unfolding, predominantly feed-forward neuronal cascades.

## Introduction

Considerable effort is currently dedicated to characterize mesoscopic dynamics of the cortex by recording simultaneously from as many neurons as possible *in vitro* [1]–[3] and *in vivo* [4]–[14]. However, except for the rare situation like imaging the full larval zebrafish brain [15], current technologies for the mammalian cortex allow only for studying relatively small parts of the full network. For example, in non-human primates, microelectrode arrays typically cover a cortical area of tens of mm^2^ [8]–[10], [12], [16], [17], which is many times smaller than the full cortical surface.

The problem of neuronal sub-sampling can be partly alleviated by studying the local field potential (LFP), which reflects synchronized activity of neuronal groups [1], [8], [17]. Yet, despite the capability to collect activities of many more neurons, the spatial scale to which the LFP can be measured is still limited by, e.g., the size of the recording array. The consequences of such ‘windowed’ observations for studying cortical dynamics are not well understood. In the present study, we investigate this problem by analyzing cortical neuronal avalanches in both ongoing LFPs from awake monkeys and neuronal network simulations.

Neuronal avalanches are spontaneous activity cascades in superficial layers of cortex that follow precise statistical relationships characterized by power laws [1], [8], [18]–[21]. The distribution of avalanche sizes follows a power law with exponent –1.5, which indicates long-range spatiotemporal correlations for which the observation is particularly affected by spatially ‘windowed’ recordings. Theory [22], [23] and experiments [24]–[26] suggest that critical dynamics, indicated by avalanche sizes that follow a power law [27], provide networks with maximal dynamic range, pattern entropy, phase variability [28] and learning capabilities [29]. Thus, the power-law distribution in avalanche size is an important indicator of critical dynamics that requires proper identification. In the current study, we identify the window-size effect in analyzing avalanche dynamics and detail how a window introduces an upper cut-off in the power-law distribution of avalanche sizes composed of a biased sub-sampling of spatiotemporal patterns. We show that properly taking the cut-off into account resolves apparent and substantial differences on assessing avalanche dynamics, such as more negative power-law exponents, as recently reported by [30].

## Materials and Methods

### Ethics statement

All experiments were carried out in accordance with the US National Institutes of Health guidelines for animal use and care. All procedures were approved by the Animal Care and Use Committee of the National Institute of Mental Health.

### Subjects and experimental procedures

Subjects for this study were two adult macaque monkeys (Macaca mulatta; one male and one female; 7–8 years old; weighting 7–9 kg). The animals were housed in individual cages in an animal room, with 12-h light/dark cycle. For environmental enrichment, toys were placed within the cages. Water was provided ad libitum and two meals were given each day. To implant the head posts and microelectrode arrays, aseptic surgeries were carried out under anesthesia (isoflurane, 1–4%) and overseen by a veterinarian. The detailed surgical procedures were described previously [31]. Recordings were carried out after sufficient recovery from the surgeries (at least 5 days, determined by a veterinarian). After experiments were finished, the head posts were removed and the skins were sutured.

### Electrophysiological recordings

In the two adult macaque monkeys, we recorded the LFP with chronically implanted microelectrode arrays (96 channels; 10×10 without corner electrodes, 400 µm inter-electrode distance, 0.5–1 mm electrode length; BlackRock Microsystems) located in the superficial layers of the arm-representing area of the left premotor cortex (monkey 1; 91 working electrodes) or prefrontal cortex (area 46; monkey 2; 95 working electrodes). 20–30 minutes of ongoing LFP (1 to 100 Hz band-pass filtered) signals were simultaneously obtained (2 kHz sampling frequency) from each electrode while the monkey was sitting awake in a primate chair with the head fixed, but not engaged in any behavioral task. For some analyses, LFP signals were filtered with a higher cut-off frequency of up to 250 Hz as indicated in the main text and figure legends. The same data set has been analysed previously [17], [31], [32].

### Avalanche analysis

**Figure 1:**
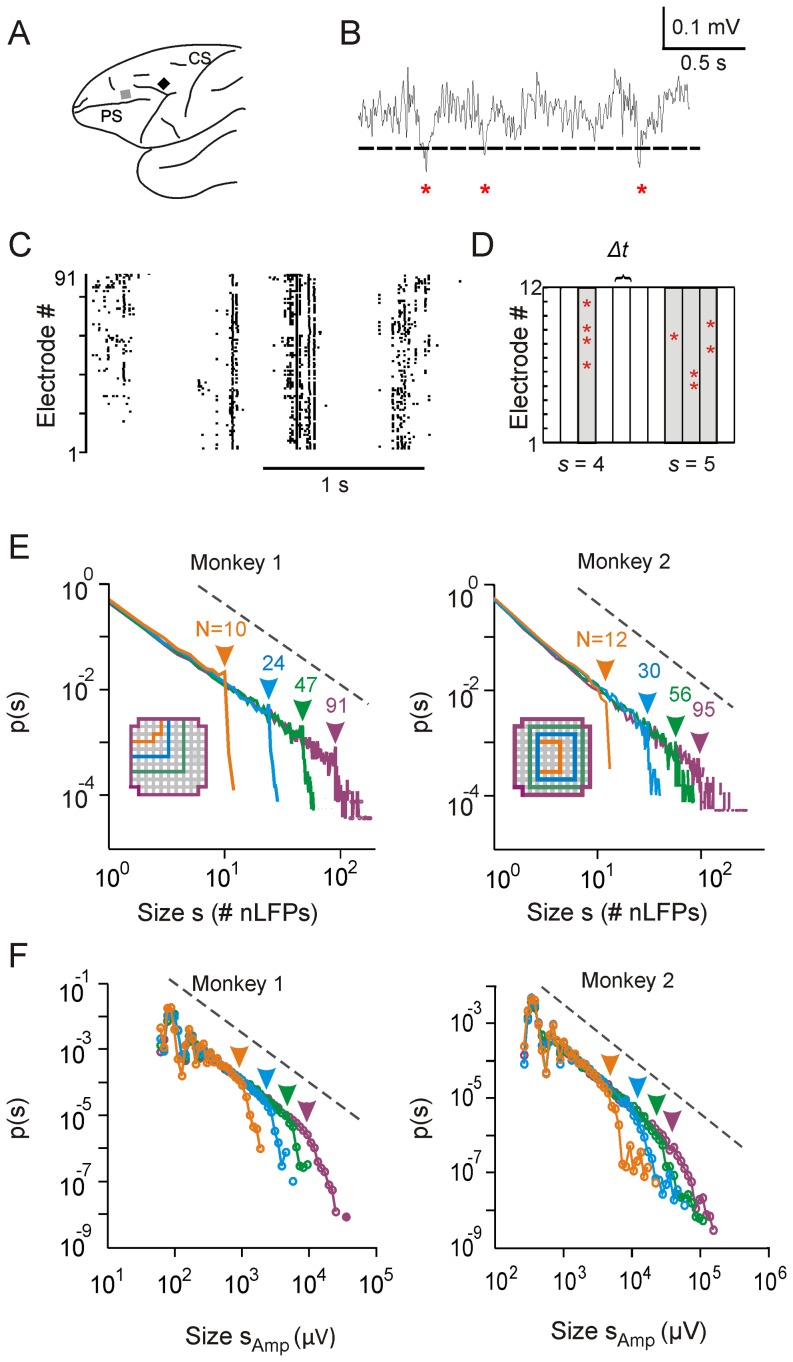
Observation window limit introduces cut-off in avalanche size distributions. (**A**) Sketch of macaque brain with microelectrode array locations (squares; black: premotor cortex, monkey 1; gray: prefrontal cortex, monkey 2). *CS*: central sulcus; *PS*: principal sulcus. (**B**) An example trace of an LFP signal, showing the detection of nLFPs (marked by asterisks) using a threshold of −2.5 SD (dashed line). (**C**) Raster plot of nLFPs detected from all 91 electrodes (monkey 1) in a period of 2 seconds. nLFPs are represented by individual dots in the plot. (**D**) nLFPs occurring during either the same or consecutive time bins are detected as a spatiotemporal cluster with the size, *s*, defined as the number of nLFPs involved. (**E**) Avalanche size distributions are plotted in double-logarithmic coordinates for four observation windows, i.e., groups of electrodes in the recording array. The size of the observation window, *N*, is defined as the number of electrodes within the window (see inset for spatial coverage of the windows). The positions of arrows indicate the values of the corresponding *N*. (**F**) Continuous avalanche size distributions are plotted for the same observation windows with the size of an avalanche, *s_Amp_*, defined as the summated absolute amplitudes of all nLFPs involved. The positions of arrows indicate the values of *N*× mean absolute nLFP amplitude across all electrodes. For visual comparison, a power law with exponent −1.5 is shown in *E* and *F* (dashed lines). *E* is re-plotted from [17], [32].

Large negative deflections in the LFP, i.e., nLFPs, were detected with a threshold of -2.5 standard deviations (SD) of the LFP fluctuations estimated separately for individual electrodes. nLFP events have been demonstrated to be associated with a significant increase of spiking activities and neuronal synchrony and therefore reflect the activity of local neuronal populations [8], [17]. The nLFP peak times were subsequently binned using a time window, *Δt* (2 ms for monkey 1; 4 ms for monkey 2; similar results such as power-law size distributions can be obtained with different bin widths, see refs. [1], [8]), to identify the cascading activities. A time bin was defined active if it contained at least one nLFP at any of the recording sites within the spatial extent of the analysis. Spatiotemporal clusters of nLFPs were then defined by nLFPs that occurred within a single time bin or within consecutive time bins, regardless of their spatial location. By definition, a cluster is always flanked by inactive bins in which no nLFP was detected (Fig. 1D). The size of a cluster, *s*, was defined as either the number of nLFPs in that cluster (discrete size) or the sum of the absolute amplitudes of all nLFPs in that cluster (continuous size). Continuous sizes were logarithmically binned. All cluster size distributions were plotted in double-logarithmic coordinates for visual inspection.

### Estimation of the branching parameter

**Figure 2:**
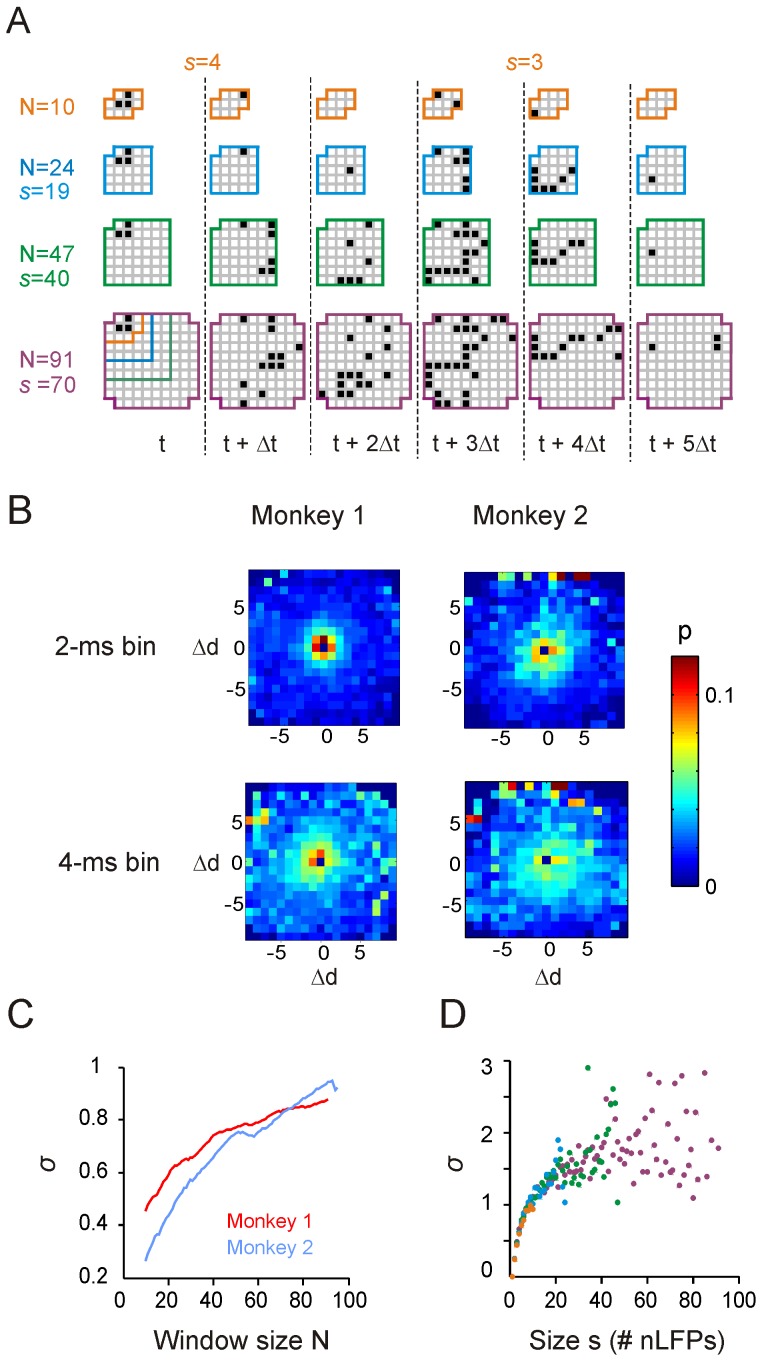
Local activity propagation leads to avalanche dynamics that can be observed by windows with varying sizes. (**A**) Small avalanches identified within small windows are parts of larger avalanches identified in large windows. Examples of the spatiotemporal pattern of an avalanche as observed through windows of increasing size. Note that for the smallest window, the avalanche was separated into two smaller avalanches. (**B**) Probability map of nLFP propagations, showing the probability, *p*, of detecting a decedent nLFP at certain location in the next time bin (2 ms, upper row; 4 ms, lower row) after a single nLFP has been detected. The initial nLFP is always positioned at the center of the map (0, 0) and the unit of distance, *Δd*, is the inter-electrode distance of the recording array (0.4 mm). (**C**) Estimation of balanced propagation depends on window size. The estimated branching parameter, *σ*, increases with window *N*, approaching the critical value of *σ* = 1. (**D**) Branching parameter as a function of avalanche size, *σ*(*s*), is plotted for the four observation windows used in *A* (color coded). Individual dots represent average *σ* for avalanches with different sizes, *s* = 1, …, *N* for monkey 1 (monkey 2 gave similar results; not shown).

The branching parameter, *σ*, is defined as the ratio between the number of active sites at time *t*+1 and the number of active sites at time *t*. For a single avalanche, *σ* can be obtained by averaging the ratio across all time bins of that avalanche. Similarly, by averaging the ratio across specific groups of avalanches, we can calculate *σ* for 1) avalanches with the same size (Fig. 2D), or 2) all avalanches observed by the same observation window (Fig. 2C). Note that in previous studies (e.g., see [1]), *σ* was calculated by looking at the same ratio but only for the first time bin in avalanches (if an avalanche lasts only for one time bin, the ratio is zero). Here we calculated the ratio for all time bins in avalanches in order to take more information about activity propagation into account. We also analyzed our data according to the previously used definition of *σ* and all conclusions held.

### Visualization and analysis of probability distributions with and without a cut-off

The fitting of a statistical model to empirical data requires both a well-motivated statistical model (power law, exponential, etc.) and a proper specification of the range of values over which the data is properly fitted by the model. The importance of the latter becomes evident when considering a power-law distribution with an upper cut-off (see below).

For the continuous power-law density function (PDF) without cut-off, *p*(*s*)  = *cs^α^*, the corresponding complementary cumulative distribution function (CCDF) is 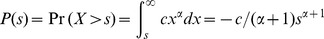, where *c* is the normalization factor. Thus, the CCDF is a power law with exponent *α*+1, which allows examining the linear relation in double-logarithmic coordinates to visually assess if an empirical distribution follows a power law [30], [33], [34]. Consequently, the exponent, *α*, of a power law without cut-off can be inferred by estimating the exponent of the corresponding CCDF. This method, however, has a caveat in the case of power laws with an upper cut-off as pointed out by others [35]. For a distribution with an upper, finite cut-off, *s*_max_, the CCDF is given by 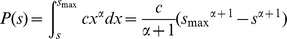. Such a function is equivalent to a power law plus a constant, and will not be a straight line in the log-log plot. Note that the above results assume *p*(*s*)  = 0 for *s*>*s*_max_ but the same conclusion holds for an arbitrary form of *p*(*s*) that deviates from the power law with exponent *α* for *s*>*s*_max_. As for continuous distributions, a cut-off in discrete power-law distributions abolishes the linear trend of the CCDF in double-logarithmic coordinates (see Results). In addition to the problems of data visualization, ignoring a cut-off in the data introduces a bias in parameter estimations (see Results). Therefore, in the current study, we identified the cut-off in the size distributions and used it to specify our statistical models accordingly [31].

### Parameter estimation

For the parameter estimation, we used a maximum likelihood approach [31], [33]. Parameter values for discrete size distributions were obtained by fitting the discrete power-law model *p_α_*(*s*) for avalanche size *s*,



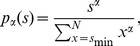
(1)to the body of the distribution, *s* = *s*_min_, …, *N*. Here, *N* denotes the array or window size and was defined as the number of electrodes used in the analysis of avalanche sizes. Using the upper bound *N* instead of infinity, prevents the parameter estimates to be affected by the cut-off in the probability distribution for *s*>*N* [31], [36], [37]. If not noted otherwise, the lower bound of the power law, *s*_min_, was set to the smallest possible cluster size *s* = 1.

The best-fit power-law exponent for a sample of *n* cluster sizes, **x** =  (*x*_1_, …, *x*_n_), can be obtained by maximizing the likelihood of the power-law model with exponent *α*,
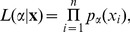
(2)or, mathematically equivalently, the logarithmically transformed likelihood
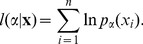
(3)


Thus, the best-fit power-law exponent 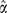 is given by
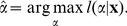
(4)


For the maximization of Eq. 3, the Nelder-Mead method was applied (using the Matlab function *fminsearch*). For a subset of distributions, the objective function in Eq. 3 was visually inspected by using a grid search over a wide parameter range (−2≤*α*≤−1) to ensure the absence of multiple, local maxima. In addition, for a subset of distributions, different initial values were tested to ensure that the algorithm would converge to the same optimum. To obtain parameter estimates for continuous size distributions, *p_α_*(*s*) in Eqs. 1-3 can be substituted by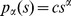, where 
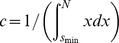
 is the normalizing constant (see also refs. [36], [37]).

### Simulation of power law distributed data

To generate power law distributed samples on the range *s* = 1, …, *N*, we applied the inverse method using the cumulative distribution 
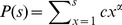
 for *s* = 1, …, *N* and *P*(*s*)  = 0 for *s* < 1. The exponent *α* is the desired power-law exponent and 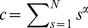 is the normalization factor. For a sample, *u*, from a uniform distribution on the interval (0, 1), the value *s* that fulfils the condition *P*(*s*-1) < *u* ≤ *P*(*s*) is a sample from the desired power-law distribution. If not stated otherwise, we used *α* = −1.5 and generated 10,000 samples for each distribution.

### Rescaling of power-law distributions

To better visualize the cut-off behavior for different window sizes, *N*, we adopted the rescaling approach described in ref. [31]. This approach collapsed power-law distributions with the same exponent for *s* ≤ *N*, and allowed for a direct visual comparison of the distribution tails (*s*>*N*). First, the power-law distribution *p*(*s*) was normalized within the range *s* = *s*_min_, …, *N*:
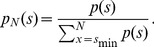
(5)


Second, dividing Eq. 5 by the the rescaling function [31],
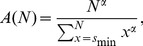
(6)results in the collapse at *p_N_* (*s*)/*A*(*N*)  = 1 and *s/N* = 1 for *s* = *N*. Note that *p*(*s*) in Eq. 5 can be an empirical distribution without or with cut-off. The only requirement for achieving a collapse is that *p*(*s*) follows a power law between *s*_min_ and *N* with exponent *α* (*α* can be estimated from the data as described above).

### Calculation of the cut-off index (CI)

To quantify the cut-off behavior of a power-law distribution *p*(*s*) for cluster sizes *s*>*N*, we defined the measure
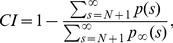
(7)where *p*(*s*) is the empirical distribution of interest, and 
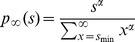
 denotes a power-law distribution without cut-off. The normalization constant for *p_∞_*(*s*) can be obtained by using the Riemann zeta function: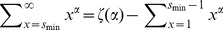. The exponent *α* used for *p*_∞_(*s*) should be estimated from the empirical distribution in the range *s* = *s*_min_, …, *N* using the likelihood estimation described above. CI is close to 0 if the empirical distribution *p*(*s*) follows a power law without a cut-off, and equal to 1 if *p*(*s*) shows a perfect cut-off beyond *N* (i.e., the probability for *s*>*N* is zero). We note that CI does not strictly range between 0 and 1 but could result in a negative value for a distribution that shows an increase in probabilities for *s*>*N* compared to a power law (this was not observed for the distributions tested in this study). The above definition of CI was not systematically affected by a change in the number of samples. In addition, the influence of varying *N* for theoretical distributions was very small, thus allowing the use of CI to compare the cut-off behaviour across different window sizes *N*.

### Two-layer network model

The network model consisted of two components: (i) a two-dimensional network of binary nodes that exhibited critical branching dynamics, and (ii) a layer of “electrodes” that represented the local spiking activity (LSA) of the underlying nodes. The neuronal network comprised 100×100 = 10,000 nodes on a grid connected with different topologies, that is, local (a node only connects to other nodes in its neighbourhood), small-world (predominantly local with some long-distance connections), and all-to-all (fully connected). If not stated otherwise, results are shown for a network with local connectivity, which resembled the average functional connectivity observed in the data (see Results). The distance between nearest-neighbour nodes (assumed to be equal to one without loss of generality) was the same along the two dimensions of the grid and was used to set the connectivity strength for the local topology. The connectivity strength, *p_ji_*, of the “postsynaptic” nodes *j* = 1, …, 10000 for a given “presynaptic” node *i* was proportional to 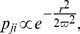
(8)where *r* denotes the Euclidian distance between nodes *i* and *j* in the two-dimensional space. To avoid dissipation of activity at borders, periodic boundary conditions were applied. We used *ω* = 4 (Eq. 8) if not stated otherwise. The resulting function corresponds to a Gaussian kernel with a half-max width of ∼9.4, resulting in ∼70 “postsynaptic” nodes that had larger than half-max connectivity strength. Connections of a neuron to itself were not considered (*p_ii_* = 0). The robustness of the presented results was confirmed in networks with small-world topology, in which 1% of connections were randomly rewired (without multiple connections between any pair of neurons and without self-connections). In addition, an all-to-all connectivity regime was tested, in which the strength of connections between neurons was drawn from a uniform distribution. As in the cases above, no self-connections were allowed. Nodes were implemented as binary units with “0” representing the inactive state and “1” representing the active state in which a spike was being generated. The postsynaptic activation of nodes was probabilistic, where *p_ji_* is the probability of node *j* being active at time step *t+*1 due to node *i* being active at time step *t*. For the simulation of a single neuronal activity cascade, the network was initialized with all nodes being inactive. An avalanche was initiated by activating a single, randomly selected node *i* at time step *t* = 1. Activity was propagated in the next time step *t*+1 according to the connectivity matrix *p_ji_*. Activity propagation was repeated until no further node was activated. An active node became inactive at the next time step if it was not activated by another node. The size of the activity cluster at the neuronal level was defined as the total number of spikes within the cluster. For each topology, between 100,000 to 150,000 avalanches were simulated. To obtain a critical branching process, the connectivity strengths were scaled to fulfil the following condition:
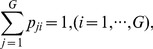
(9)where *G* = 10,000 denotes the number of nodes in the network. We note that the above activation rules and the condition in Eq. 8 can lead to a significant amount of dissipation (i.e., a node was activated by multiple inputs) if each node only connects to a few nodes in the neighborhood (e.g., for networks with 4 connections per node). For the local topology as defined above, the effect of dissipation was sufficiently small (less than 0.8% of activations resulted in multiple activations). Thus, the mean number of descendents for each active node was very close to one in the next time step. Consequently, the distribution of avalanches sizes at the neuronal level followed a power law with exponent −1.5 (see Results).

For calculation of the LSA, we created an array of 10×10 = 100 “electrodes”. Each “electrode” sampled the summed spiking activity from a field of 10×10 = 100 adjacent, non-overlapping nodes for each time step. The raw LSA varied between 0 and 100 (the number of sampled nodes per “electrode”). For some simulations, additional configurations were tested to confirm the robustness of the presented modeling results: (i) overlapping sampling of nodes, and (ii) increased number of “electrodes” (up to 625 with non-overlapping sampling of 4×4 = 16 nodes each). Similar to the temporal filtering of the LFP in the data, the raw LSA was temporally smoothed using a Gaussian function with a varying half-max width *w_h_* = 1, …, 40 time steps. For the detection of spatiotemporal clusters in the LSA, we applied a threshold *z* = 0.1 and determined the time stamp of the positive maxima above threshold, which is equivalent to detecting the negative peak below a certain threshold in the LFP. The definition of avalanches was the same as described above for the LFP activity (bin size: 8 time steps). We note that different choices of the temporal smoothing parameters and the threshold value (up to 5 times of the above value) did not change the power-law scaling of avalanche sizes *s* ≤ *N* (*N* denotes the number of simulated electrodes in the model). Results are shown for *w_h_* = 10, …, 30.

## Results

### Predominantly local propagation of neuronal activities underlies scale-invariant avalanche dynamics

When recording neuronal activity in the brain, the absolute dimension of a neuronal event that can be measured, such as the size or spatial extent of a synchronized neuronal population, is limited by the number of neurons or cortical sites recorded from or, in general, the size of the observation window. Despite this uncertainty in the absolute size of neuronal events, relative size relationships between events can still be obtained, which allows for the identification of scale-free dynamics in neuronal avalanches [1], [31], [38]. This is demonstrated in figure 1 for ongoing LFP activity (1–100 Hz; 20–30 min) recorded with microelectrode arrays (∼10×10 spatial configuration; 400 µm inter-electrode distance) in premotor and prefrontal cortex of two awake macaque monkeys sitting in a primate chair not engaged in any particular task (Fig. 1A). From the ongoing LFP, transient negative deflections (nLFPs) at individual electrodes, indicative of local synchronized activity [8], [17], were detected by thresholding (–2.5 SD; Fig. 1B). The resulting spatiotemporal organization of nLFPs among all recording sites appeared rather complex (Fig. 1C). Yet, when nLFPs on the array were concatenated into spatiotemporal clusters (Fig. 1D, see Materials and Methods), the cluster size, *s*, defined as the number of nLFPs involved, followed a fairly simple probability distribution. This probability distribution revealed a linear relationship in double-logarithmic coordinates up to the size of the entire electrode array and an abrupt drop, i.e., cut-off, beyond the array size (Fig. 1E, purple). The linear part of this distribution demonstrates that the relative occurrence for clusters of different sizes (i.e., the ratio of their respective probabilities) is constant. For example, clusters of size *s* = 1 versus *s* = 5 occur as often as clusters of size *s* = 10 versus *s* = 50. This constancy in size relationship is indicative of a power law *p*(*s*) ∼ *s^α^* here with an exponent *α* close to -1.5, the hallmark of neuronal avalanches [1], [8], [18], [20], [21], [39]. The power-law scaling before the cut-off does not depend on the size of the observation window, which is defined as the number of electrodes *N* of compact sub-arrays used for the detection of nLFP clusters (Fig. 1E). In contrast, the location of the cut-off changes systematically with *N* (Fig. 1E, shown for *N* = 10 electrodes to the whole array with *N*>90 electrodes). Similar scaling is found for continuous avalanche size probability distributions in which the size of nLFP clusters is defined as the sum of absolute nLFP peak amplitudes (Fig. 1F) [24]–[26].

The invariance of the power law to observation window size demonstrates a specific underlying organization of avalanches as exemplified in figure 2A, where avalanches in a given time period were plotted for different window sizes. As expected, avalanches observed in the small window are part of larger avalanches identified in the bigger window, demonstrating that there is no certainty as to the absolute size of an observed avalanche. In principle, any observed avalanche could have originated within and remained confined to the observation window, could have migrated to the window from cortical regions outside the window, or could have left and revisited the cortical region covered by the array (i.e., window) multiple times (Fig. 2A). Given that the observed avalanche size varies with window size *N*, how can it be that the probability distribution before the cut-off in figure 1E and F is invariant to *N*? The answer is related to the propagation of nLFPs in the cortical network. Fig. 2B shows the propagation profile for near future nLFPs, i.e., nLFPs occurring at time *t_0_*+*Δt*, given an nLFP at *t_0_*. This spatial probability profile is largely local; on average, the next nLFP in an avalanche is likely to be spatially close to the current nLFP. Thus, as long as the window of observation is large enough to monitor such local propagation, the size relationship of the observed avalanches will reflect the scale-invariant organization of the underlying dynamics.

Neuronal avalanches have been originally introduced with the hallmarks of a power law in sizes with slope of −1.5 and a critical branching parameter *σ* = 1 [1]. The branching parameter estimates how many descendent sites will be activated given a certain number of ancestors. When *σ* = 1, on average, each nLFP at time *t* should spawn 1 nLFP at *t* + *Δt*. The predominant local propagation as shown in figure 2B might initially suggest that the estimation of *σ* does not exhibit finite-size effects. However, as shown in figure 2C, *σ* increases with window size *N*, approaching the value of *σ* = 1 for large *N*. To clarify this dependency of *σ* on *N*, we calculated *σ* as a function of avalanche size *s* for 4 different window sizes. Figure 2D demonstrates that *σ*(*s*) increases with *s*, which can be understood intuitively when one recognizes that activity propagation is a probabilistic process. That is, large avalanches happen to produce more descendants than ancestors during their initial unfolding thereby increasing their continued survival. The converse is true for small avalanches. Importantly, the relation *σ*(*s*) is found to be independent of the observation window size *N* (Fig. 2D). Therefore, the branching ratio *σ* tends to be underestimated for small windows given the cut-off in the avalanche size distributions for *s >N*, but recovers towards *σ* = 1 with increase in *N*.

### Dynamical feed-forward propagation characterizes neuronal avalanches

**Figure 3:**
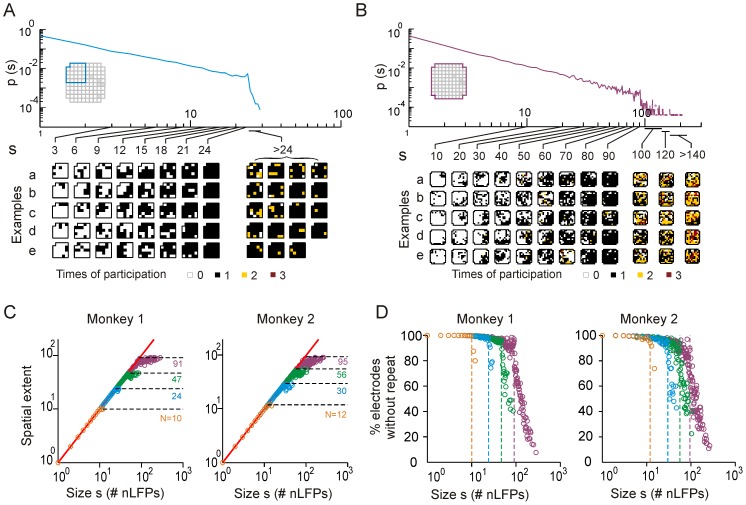
Characteristics of spatial patterns for avalanches observed before and after the cut-off. (**A**) Avalanches observed with a window of *N* = 24 (see inset). Top panel: the probability distribution is redrawn from Fig. 1E (Monkey 1). Bottom panel: five randomly chosen spatial avalanche patterns each are shown for *s* = 3, 6, …, 24. In addition, all 19 spatial patterns for avalanches larger than the observation window size (i.e., *s*>24) are depicted. The number of times that any specific electrode participated in a given avalanche is color-coded. (**B**) Same as *A* for the largest observation window with *N* = 91 electrodes. Only 15 example patterns with s>91 are depicted. (**C**) The average spatial extent of avalanches, quantified by the number of unique electrodes involved in an avalanche, is plotted as a function of avalanche size for different observation windows. Horizontal dashed lines indicate window size *N*. The diagonal red line indicates equality. (**D**) Average percentage of electrodes that do not exhibit repeated activation in an avalanche is plotted as a function of the avalanche size for different observation windows. Vertical dashed lines correspond to the different observation window sizes. The observation windows used are the same as those in Figure 1E.

The cut-off in avalanche sizes, while not part of the power law, details additional aspects of avalanche dynamics. In fact, the size distribution plotted in figure 1 for fixed window size results from two different counting strategies imposed by the window size. This is demonstrated by re-plotting figure 1E for two window sizes of *N* = 24 and 91 electrodes, respectively, and displaying the spatial pattern of randomly selected avalanches of sizes below and above *N* (Fig. 3A and B). Repeated activations of individual sites are common for avalanches with *s*>*N*, whereas they are relatively rare for *s* ≤ *N*, which is quantified further in figure 3C and D. Fig. 3C demonstrates that the cluster size for *s* ≤ *N* largely reflects the spatial extent of an avalanche. This is clearly shown by all points in Fig. 3C falling on or close to the diagonal line up to *N*, whereas data differ for s>*N*. We note that, if avalanches were dominated by repeated activations of sites, individual avalanche sizes would scatter within the area below the diagonal. Instead, these plots demonstrate the general lacking of repeated activations in avalanche propagation, that is, the set of active sites at time *t* is not likely to be revisited as the avalanche unfolds. We refer to this as dynamical feed-forward propagation of avalanche activity. In contrast, avalanches with *s*>*N* are composed of activity that spans nearly the whole observation window in addition to repeated activations of many recording sites, resulting in a rapid decrease of the percentage of non-repeating electrodes (Fig. 3D). The apparent over-abundance of repeats is not a true aspect of avalanche dynamics, but instead arises from highly biased counting due to the inability of observing non-repeated patterns of *s*>*N*. Accordingly, the small percentage of non-repeating electrodes measured for a given size, *s*, beyond the cut-off with a smaller window is no longer observed for the same size *s* when observed with a larger window [e.g., compare any size from *s* = 60 to70 for *N* = 47 (green) with the same size for *N* = 91 (purple) for monkey 1 in Fig. 3D]. Thus, the pattern space for sizes below and beyond the observation window size differs, which is based on two different counting schemes. Up to the cut-off, avalanche sizes can be realized by non-repeated as well as repeated activations, whereas beyond the cut-off, avalanche sizes can only be realized with repeated activations. Accordingly, each avalanche size distribution in figure1E and 3A and B in fact is composed of two different distributions separated by the window size *N*.

### A two-layer neuron-electrode model of avalanche dynamics captures feed-forward propagation and size cut-off

**Figure 4:**
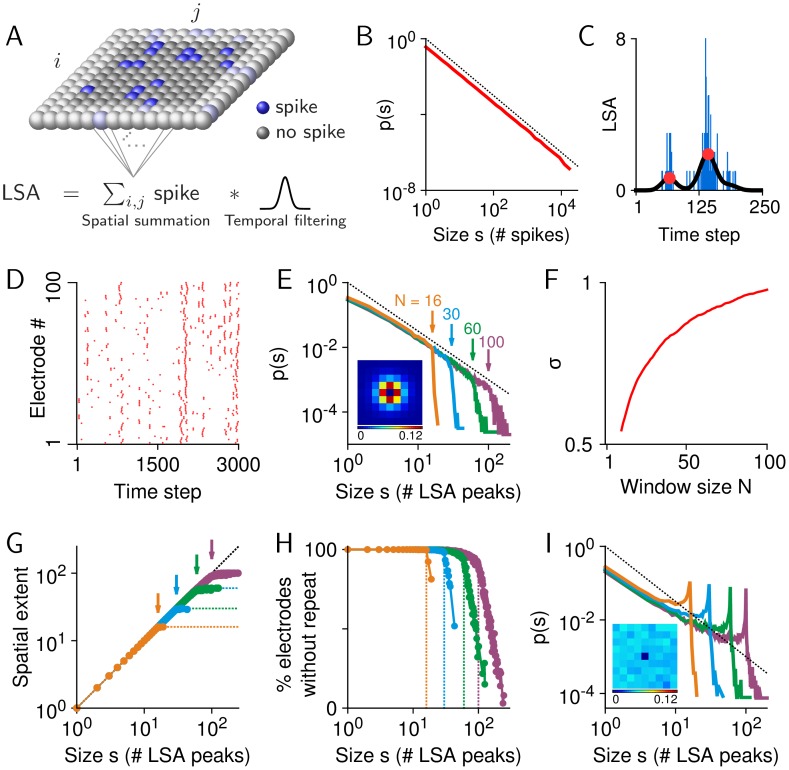
The two-layer model exhibits dynamics similar to LFP-based cortical neuronal avalanches. (**A**) The diagram of the model, showing a part of the two-dimensional network of binary neurons and the generation of signals at the “electrode level”, i.e., the local spiking activity (LSA). The LSA sampled by simulated electrodes is produced by summation of spiking activities from spatially compact, non-overlapping 10 by 10 neuronal groups (dark gray and blue nodes) and subsequent temporal smoothing. (**B**) The size distribution of spike avalanches (*n* = 150,000; red) in the critically tuned network follows a power law with exponent −1.5 (dashed line). (**C**) Example trace of raw (blue) and temporally smoothed (black) LSA activities (half-width of the Gaussian smoothing window: 30 time steps). LSA peaks (red dots) were detected by applying a threshold of LSA = 0.1. (**D**) Raster of LSA peaks detected at the electrode level (individual dots represent LSA peaks). (**E**) Avalanche size distributions observed at the electrode level of the model with local connectivity are plotted for four different observation windows (*n* = 50,105 avalanches for *N* = 100). Inset: probability of LSA propagation across the two-dimensional array of simulated electrodes. The positions of arrows indicate the corresponding window sizes. The dotted line is a power law with exponent of −1.5. (**F**) The estimated branching parameter, *σ*, is plotted against the observation window size *N*. (**G**) The average spatial extent, quantified by the number of unique electrodes involved in an avalanche, is plotted against avalanche size for different observation windows. The horizontal dotted lines indicate window sizes (same as *E*). The diagonal dotted line indicates equality. (**H**) Percentage of electrodes without repeated activation during an avalanche is plotted as a function of avalanche size. (**I**) The same as in *E* for all-to-all connectivity (inset shows the probability of LSA propagation across the electrodes).

Further insights into these experimental findings were obtained using a 100×100 network of binary neurons (Fig. 4A) placed on a grid with local connectivity that approximated the spatial unfolding of cortical avalanches (Fig. 2B). Spike propagation in the model was tuned to be critical, that is, on average, one neuron excited one neuron in the next time step (see Materials and Methods). Accordingly, the size of spike avalanches followed a power-law probability distribution with slope of −1.5 (Fig. 4B, see also [40]). To simulate population activity in a layer of 100 electrodes, local spiking activity (LSA) was summed from non-overlapping, compact 10×10 groups of binary neurons and temporally smoothed (Fig. 4A and C). Suprathreshold, positive LSA maxima (LSA ≥ 0.1; Fig. 4C, red dots) identified local transients in population synchrony similar to nLFPs.

For the local connectivity regime (Fig. 4E, inset), the LSA raster (Fig. 4D) exhibited the spatiotemporal organization of avalanche activities, which distributed in size according to a power law with slope of −1.5 for *s* ≤ *N* and exhibited a cut-off for *s*>*N* (Fig. 4E). Similar to the result observed for the empirical data, we found that the estimated branching parameter for LSA cascades depended on window size *N* (Fig. 4F), although the model was tuned to be critical and the mean number of descendants of each active node was equal to one at the neuronal level. Importantly, our simulations also demonstrated dynamic feed-forward propagation of local group activity within avalanches for *s* ≤ *N*, i.e., the avalanche size reflects its spatial extent with few reactivations of individual electrodes. In contrast, reactivation was prevalent for *s*>*N* (Fig. 4G and H). In the absence of external input to the simulated neuronal network, these reactivations reflect the re-recruitment of sites active earlier in an avalanche as the avalanche unfolds. The power law with exponent −1.5 was also observed in a network with small-world topology (with ∼1% long-range connections; data not shown), which is in line with the previous finding of the coexistence of small-world topology and avalanche dynamics in the cortex [41]. In contrast, a network with all-to-all connections produced distributions with a shallower initial part and a cut-off that was characterized by a pronounced peak (Fig. 4I). We point out that the distribution of avalanche sizes at the neuronal level for the all-to-all connectivity is well described by a power law with exponent −1.5 (similar to the local connectivity as shown in Fig. 4B). Therefore, the local propagation of LSA activities, which is consistent with the LFP results, was required to achieve the scale invariance of the power-law distributions and the cut-off as shown in figure 1.

### The cut-off in size distribution depends on sampling parameters

**Figure 5:**
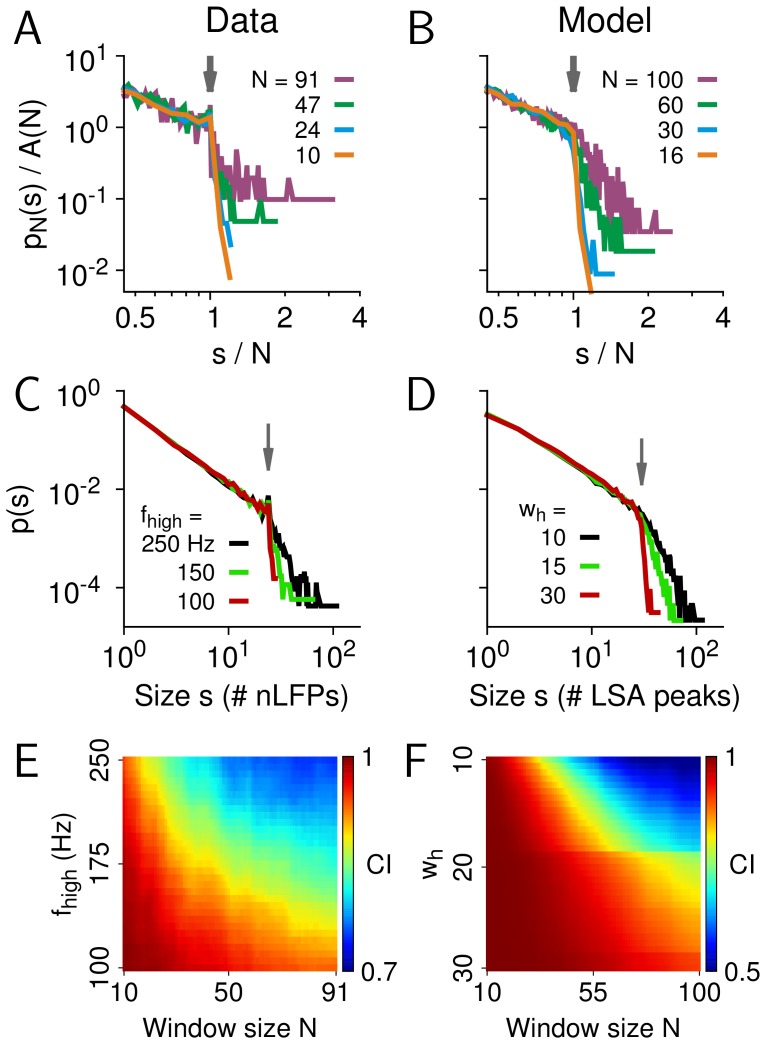
Quantification of the cut-off. (**A**) Rescaled cluster size distributions for monkey 1 show the collapse of the distributions before *s/N* = 1 (vertical arrow) and different cut-off behaviour for *s/N*>1 for four different array sizes. (**B**) The same as *A* for the model. (**C**) Cut-off behaviour of cluster size distributions that were obtained for different temporal filter settings. Data was filtered with the same lower cut-off frequency (1 Hz) but different upper cut-off frequencies, *f_high_* = 100, 150, and 250 Hz (monkey 1; vertical arrow indicates the array size: *N* = 24 electrodes). (**D**) The same for the model. Raw LSA traces were smoothed with Gaussian filters of various half-widths (*w_h_* = 10, 15, and 30 time steps; array size: *N* = 30 electrodes). (**E**) Cut-off index CI (Eq. 7) for size distributions in monkey 1 for all combinations of *f_high_* and *N*. (**F**) CI for distributions obtained from the model with different values of temporal smoothing (*w_h_*) and *N*. To estimate *α* for the calculation of CI for the model, *s*_min_ = 4 was used.

We found that the steepness of the cut-off changed with the observation window size for both data and the model. To allow for a direct visual comparison of the cut-offs, we calculated rescaled cluster size distributions that resulted in a collapse of the distributions for *s* ≤ *N*. As shown in Fig. 5A and B for the data and model, respectively, smaller window sizes have stronger (steeper) cut-offs. The fact that the same qualitative behavior was found in both data and our model tuned to be critical suggests that such a relation between the steepness of cut-off and the window size *N* is a result of observing critical avalanche dynamics with a limited observation window.

In addition, the cut-off was affected by how quickly a given site was reactivated, which is related to the frequency components in the LFP signals. Consequently, signals with more high-frequency components tended to have a less steep cut-off for relevant frequency ranges of the LFP signal, i.e., below 250 Hz (Fig. 5C). As expected for scale-invariant dynamics, the avalanche size distributions for *s* ≤ *N* were invariant to changes in signal frequency components. This behavior was also replicated in our model using temporal smoothing kernels with decreasing widths (Fig. 5D). The impression given by these examples can be substantiated by quantitative analysis. To this end, we used the cut-off index, CI, to characterize how strong the cut-off is. CI is 1 for size distributions with a complete cut-off (probabilities for *s*>*N* are strictly zero) and is smaller than 1 for size distributions with a less pronounced cut-off (see Materials and Methods for details). Figure 5E summarizes for data from monkey 1 how the window size and frequency components of the signal jointly affect the steepness of the cut-off. In essence, the cut-off is most pronounced for small observation windows and signals containing less high-frequency components. Importantly, the same behavior can be observed for our critical model (Fig. 5F).

### The cut-off is not predictive of avalanche size relationships

**Figure 6:**
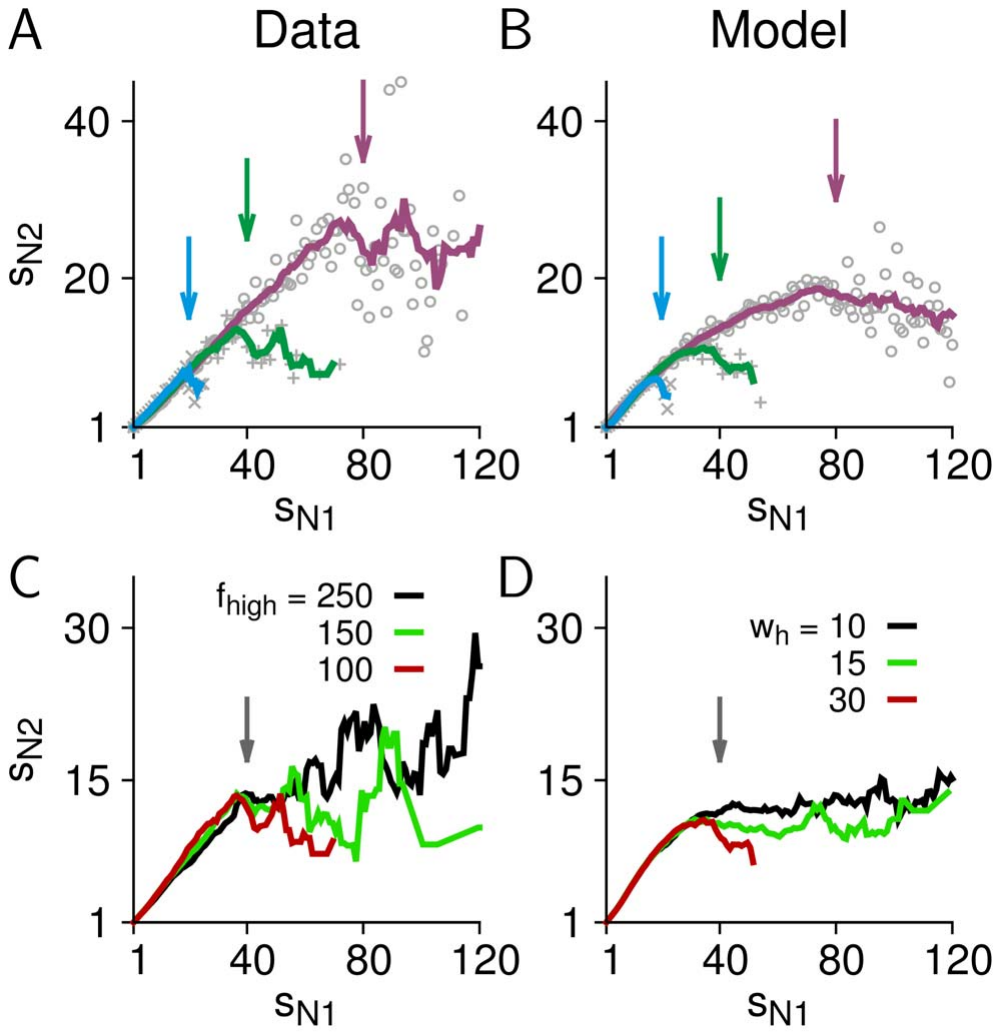
Size relationship of avalanches is only preserved for avalanches smaller than the observation window size. (**A**) Observing an avalanche of size *s_N1_ ≤ N_1_* predicts the size *s_N2_* of the corresponding avalanche observed in window *N_2_ < N_1_*. This prediction power is lost for *s_N1_*>*N_1_*. The sizes of nLFP clusters were measured for a window of size *N*_1_ and plotted against the corresponding cluster sizes that were obtained for a window half as large, i.e., *N*_2_ = 0.5×*N*_1_ (monkey 1). Vertical arrows indicate the sizes of the larger window. Shown are averages for each size *s_N_*_1_ (gray symbols) and smoothed lines for better visualization (×: *N*_1_ = 20, +: *N*_1_ = 40, o: *N*_1_ = 80). The smaller window with *N*_2_ electrodes was completely contained within the larger window with *N*_1_ electrodes. (**B**) The same as *A* for the model. (**C**) The same analysis for various values of the upper cut-off frequency, *f*_high_ (*N*_1_ = 40, *N*_2_ = 20; monkey 1). (**D**) The same as *C* for the model with various settings for temporal smoothing of the raw LSA signal.

As shown above, cascade sizes beyond the cut-off do not reflect the scale-invariant properties of neuronal avalanches. The lack of such scale-invariance, however, does not exclude the possibility that cascades in the cut-off are predictive of cascade sizes within sub-regions of the array, thus reflecting a size relationship across different observation windows. For all avalanches before the cut-off, we found a positive relation between sizes *s_N_*_1_ and *s_N_*_2,_ obtained by using two windows of size *N_1_* and *N_2_* (*N_2_ < N_1_*), respectively, to observe the corresponding avalanches. However, this relationship breaks down for *s_N_*_1_>*N*_1_ as observed in the data and the model (Fig. 6A and B, respectively). In addition, this breakdown can be observed with different upper cut-off frequencies used for filtering the LFP (Fig. 6C) or temporal smoothing settings used in the simulation (Fig. 6D). Thus, observing a pattern of size *s_N_*_1_>*N*_1_ does not predict the size of the corresponding pattern observed within a smaller spatial window of size *N*_2_. These results demonstrate that neuronal cascades with sizes beyond the cut-off contain limited to no information about the scale-invariant organization of avalanches.

### The impact of the cut-off on estimating avalanche parameters

**Figure 7:**
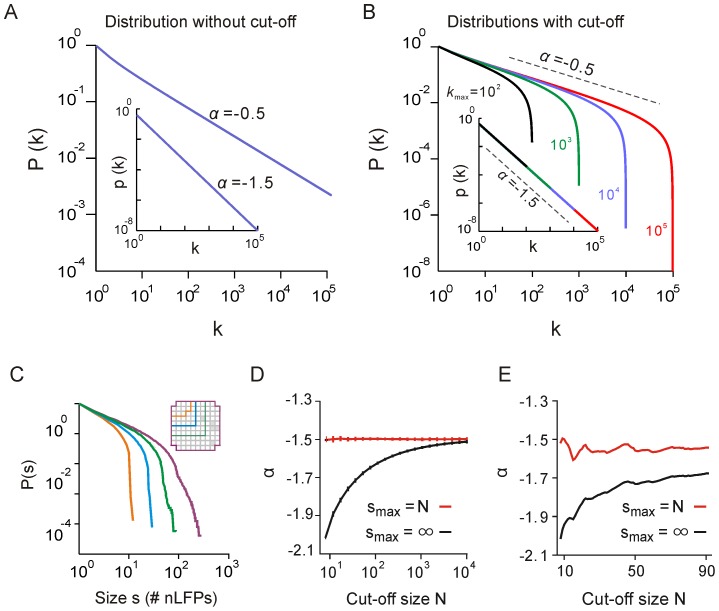
The impact of a cut-off on visualizing a power-law distribution and estimating the exponent. (A) Probability mass function (PMF, inset; calculated as 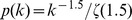, where 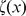 is the Riemann zeta function) and the corresponding complementary cumulative distribution [CCDF, defined as 
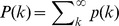
] for a power-law distribution without cut-off, i.e., the power law holds for arbitrarily large *k*. The exponents are −1.5 and −0.5 for the PMF and CCDF, respectively. (B) PMFs (inset; defined as 
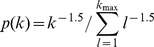
 if *k* ≤ *k_max_* and 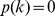 if *k*>*k_max_*, where *k_max_* is the cut-off size) and corresponding CCDFs [defined as 
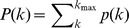
] for power-law distributions with cut-off sizes, *k_max_* = 10^2^, 10^3^, 10^4^, and 10^5^ [dashed lines: power law with exponent *α* = −1.5 (inset) and −0.5 shown for comparison]. (C) CCDFs for cluster sizes in monkey 1 (see Fig. 1E for corresponding PMFs). (D) Power-law exponents were estimated for synthetic data with varying cut-off size, *N*, ranging from 8 to 10^4^, assuming the correct model with upper bound (*s*_max_ = *N*, red) or an incorrect model without cut-off (*s*_max_ = ∞, black). Exponents were estimated using a maximum-likelihood approach (shown are the means with error bars indicating the standard deviation across *n* = 10 synthetic distributions). (E) Power-law exponents were estimated for size distribution of monkey 1 with varying cut-off size, *N*, ranging from 10 to 91, assuming the correct model with upper bound (*s*_max_ = *N*, red) or an incorrect model without cut-off (*s*_max_ = ∞, black).

Finally, we quantified how the failure to identify a cut-off in avalanche size distributions affects the examination and interpretation of potential power-law behaviour. For power-law distributions without a cut-off, i.e., *p* ∼ *s* ^α^ is valid for arbitrarily large *s*, it has been suggested that, instead of studying the probability function of the distribution, it might be beneficial to study the corresponding cumulative distribution function (CDF) or its complementary form (CCDF) [34]. The benefits stem from the fact that both CDF and CCDF do not require binning and are relatively robust against sampling noise. For the case without cut-off, the CCDF follows a power law with exponent *α*+1 (Fig. 7A). However, if the power-law probability density/mass function (PDF/PMF) has a cut-off, i.e., the power-law relationship does not hold for *s*→∞, the corresponding CCDF will not be a simple power law [35], and consequently, will not exhibit a linear relation in double-logarithmic plots (see Material and Methods for details). We simulated four power-law PMFs with various cut-offs (*k*_max_ = 10^2^, 10^3^, 10^4^, 10^5^) and plotted the PMFs and corresponding CCDFs in figure 7B. The linear relation in the PMFs was abolished in the corresponding CCDFs. Although the approximate linear regime in the CCDFs enlarges with the increase in cut-off location, no clear linear regime can be found for *k*_max_ ∼100, which is the upper size for most currently available microelectrode arrays (Fig. 7B, black). To illustrate how this can affect the examination of actual neuronal avalanches, we re-plotted the CCDFs for the avalanche size distributions for the data (Fig. 7C; monkey 1). Clearly, although the linear relation is striking in the PMFs (*cf.* Fig. 1E), it no longer exists in the corresponding CCDFs. We conclude that, if visual examination is used as a first step to evaluate the power-law hypothesis, the PMF is more informative than the CCDF. Note that Fig. 7A and B illustrate the case for discrete distributions. The same conclusion, that is, a power law distribution with a cut-off will lead to a curved CCDF in log-log plot, holds true also for continuous distributions (see Materials and Methods).

A cut-off in the empirical distribution will also affect the estimation of the power-law exponent using statistical models. We estimated the exponent *α* of a synthetic power law with cut-off by using maximum likelihood estimation with a model that does not take the cut-off into account. Figure 7D (black line) shows that ignoring the cut-off results in more negative estimates of *α* (the same behavior was observed when estimating *α* using the Kolmogorov-Smirnov statistic; data not shown). Intuitively, this is because a more negative exponent is required to explain the abrupt drop in probabilities beyond the cut-off. The bias is particularly pronounced for smaller cut-off sizes, *N*, and is more tolerable for *N*>10^3^ (Fig. 7D, black). In contrast, taking the cut-off into account results in the correct estimation of *α* in both the model and data (Fig. 7D and E, red).

## Discussion

This study demonstrates that limitations imposed by a small observation window to study large neuronal populations can be overcome for neuronal avalanche dynamics. Based on ongoing cortical activity from awake, non-human primates at rest and neuronal modeling, we show that proper size relationships of neuronal avalanches are obtained within the window of observation despite the general uncertainty about the absolute size of the population events, i.e., avalanches. Using neuronal modeling, we show that this result is a consequence of both critical dynamics and local activity propagation. By comparing our results obtained with observation windows of different sizes, we identify the unbiased regime of measurements to identify avalanche dynamics and demonstrate its qualitative separation from a sampling-biased regime identified by the size cut-off beyond the window size. Ignoring the cut-off in avalanche size distributions leads to false estimates of avalanche properties, e.g., the exponent of the avalanche size distribution. In contrast, the proper identification of the cut-off allowed us to demonstrate the dynamical feed-forward property of avalanche propagation.

### The effects of observation window on examining and interpreting neuronal avalanches

A major motivation for the current study was to identify the influence imposed by observing neuronal dynamics through a relatively small spatial window. For neuronal avalanches, we found that such windowed access indeed affects the visualization and analysis of the data, as well as the interpretation of the results. Specifically, we found that the spatiotemporal clusters with *s*>*N* were affected by sampling parameters and failed to reflect the scale-invariant properties of the underlying dynamics. Therefore, the cut-off in size distributions needs to be identified and to be excluded for a proper analysis of the data and interpretation of the results. Firstly, an upper limit in size (*s_max_*) that corresponds to the cut-off is necessary when formulating a statistical model to fit size distributions as suggested previously [31], [36], [37]. Secondly, if a cut-off is present in the probability distribution, one cannot judge if the distribution follows a power law by visually inspecting the corresponding CCDF [35]. Recently, Dehghani et al. [30] reported for recordings in cats, monkeys and human subjects curved CCDFs in log-log plots as well as more negative exponents (e.g. −3 to −4) in cluster size distributions, which was interpreted as a lack of avalanche dynamics. Closer examination of their figures, however, revealed that clusters with *s*>*N* were not excluded and many estimations were exclusively based on clusters with *s*>*N* for which fits with steep slopes are to be expected. In line with our analysis, Dehghani et al. noticed that in many of their cases, the PMFs exhibited linearity in log-log plots with a steep slope beyond system size and a rather curved appearance of the corresponding CCDFs.

In summary, visual inspection of the PMF or PDF is a useful first step to select the proper model (e.g., with or without a cut-off) in order to apply a more quantitative approach to examine power-law distributions.

### Features and limitations of the two-layer network model

Neuronal avalanches have been studied in networks of binary neurons (e.g. see [24]) or integrate-and-fire neurons with short-term synaptic depression [42], [43]. However, these models do not exhibit the typical, sharp cut-off behavior found in neuronal avalanches from LFP recordings. In the current study, local summation and temporal filtering of simulated, critical spiking activity at the electrode level combined with a predominantly local connectivity scheme replicated the scale invariance of the power-law body as well as the cut-off in the tail of the distribution. Although this simple mechanism could account for many statistical features observed in the data, other factors that control the specific shape of the distribution that constitutes the cut-off might also exist. For example, non-specific inhibition in cortical networks could create temporal windows of decreased excitability and consequently lead to a reduced likelihood of repeated activation of a cortical site during an avalanche. In addition, refractory periods that are well-known to influence spiking at the cellular level, might also influence dynamics at the population level. It should be noted, however, that the implementation of a simple refractory period does not lead to a sharp cut-off in spike avalanche distributions, but instead results in distributions with an exponential cut-off caused by the dissipation of input to neurons during their refractory periods [44].

Despite the simplicity of our model and the fact that not all aspects were precisely tuned to match the data, e.g., exact functional connectivity or exact match of the time step for spike propagation, the model of simulated LSA activities was able to correctly replicate major aspects of cortical neuronal avalanches. Importantly, the model allowed us to study neuronal avalanches in the absence of any external inputs and volume conduction. Highly consistent results obtained in both the monkey recordings and the simulations suggest that the nLFP recordings were not affected by external noise but instead reflect the intrinsic organization of neuronal dynamics.

### Predominantly local feed-forward activity propagation of neuronal avalanches

In the current study, we extracted both local and global aspects of avalanche dynamics. The local, average activity profile that characterizes the spatial propagation of successive nLFPs within an avalanche is consistent with the general notion that neuronal activity in the recurrent cortical network can propagate in a local and feed-forward manner. Using LFP or voltage sensitive dye signals, spontaneous activity in many cortical areas, including the visual, auditory, somatosensory, motor and premotor cortices is often characterized as wave-like [9], [45]–[48]. The feed-forward propagation may reflect the distributed nature of cortical operation [49], such as the maintenance of memory in recurrently connected circuits [50] and selective signal amplification by hidden feed-forward connectivity between activity patterns [51]. Moreover, our finding about the dominantly feed-forward propagation of avalanche activities is also consistent with previous suggestion that avalanches can arise from cascading activities unfolding in networks of functionally feed-forward connections [52], [53]. It is worth noting that although avalanche propagation was found to be dominantly feed-forward, avalanche dynamics does not establish exclusively feed-forward propagation [53]. Importantly, with increasing observation window size, more repeat activations can be detected even for *s* < *N*. One possible explanation is that with the increased observation window, longer lasting avalanches, which allow more time for sites to reactivate, can be detected. Thus, feed-forward propagation characterizes the local evolution of avalanches in both space and time.

Our study, besides demonstrating local feed-forward propagation for avalanche dynamics, in addition, also identifies the regime in which these local dynamics operate, which has direct implication for the global aspect of these dynamics. Specifically, if feed-forward propagation was subcritical and quickly died out, activation initiated within the observation window would not spread far and therefore would allow for a fairly complete observation using a window of limited size. Similarly, if propagation was supercritical and always engaged the majority of sites in the system, a window of limited size would also suffice, because the entire system is synchronized and observing a part of it would be predictive of the full system dynamics. In between these extreme scenarios, local propagation of activities, in principle, can lead to cascades of all possible sizes, in which case a limited observation window will be insufficient to capture the complete activity patterns. Specifically, in the critical state, the variability in cascade sizes is maximized [25], quantified by the scale-free power law in pattern size distribution, and, accordingly, the difficulty in predicting the complete pattern. The empirical data used in the current study was previously shown to be in line with critical state dynamics [32], in which long-range correlations that extend beyond the size of limited observation windows exist [54]. Moreover, the model we simulated in the current study was tuned to be critical. Despite the absence of information about the full activity patterns, we found that, in the critical state, a limited observation window can, nevertheless, correctly reflect the true organization of the underlying dynamics if the activity propagation is predominantly local. This was demonstrated by the invariant power law across different observation windows for both the data and the model with critical dynamics.

In summary, our results show that the observation window effect, i.e., the cut-off, can be identified for avalanche dynamics and, when taken properly into account, reveals a dominant local feed-forward propagation that underlies the scale-invariant organization of cortical neuronal avalanches.
